# High-Glucose or -Fructose Diet Cause Changes of the Gut Microbiota and Metabolic Disorders in Mice without Body Weight Change

**DOI:** 10.3390/nu10060761

**Published:** 2018-06-13

**Authors:** Moon Ho Do, Eunjung Lee, Mi-Jin Oh, Yoonsook Kim, Ho-Young Park

**Affiliations:** 1 Research Division of Food Functionality, Korea Food Research Institute, Jeollabuk-do 55365, Korea; Do.Moon-ho@kfri.re.kr (M.H.D.); Oh.Mi-jin@kfri.re.kr (M.-J.O.); kimyus@kfri.re.kr (Y.K.); 2 Research Division of Strategic Food Technology, Korea Food Research Institute, Jeollabuk-do 55365, Korea; ejlee@kfri.re.kr

**Keywords:** gut microbiota, high glucose diet, high fructose diet, inflammation, lipid metabolism

## Abstract

High fat diet-induced changes in gut microbiota have been linked to intestinal permeability and metabolic endotoxemia, which is related to metabolic disorders. However, the influence of a high-glucose (HGD) or high-fructose (HFrD) diet on gut microbiota is largely unknown. We performed changes of gut microbiota in HGD- or HFrD-fed C57BL/6J mice by 16S rRNA analysis. Gut microbiota-derived endotoxin-induced metabolic disorders were evaluated by glucose and insulin tolerance test, gut permeability, Western blot and histological analysis. We found that the HGD and HFrD groups had comparatively higher blood glucose and endotoxin levels, fat mass, dyslipidemia, and glucose intolerance without changes in bodyweight. The HGD- and HFrD-fed mice lost gut microbial diversity, characterized by a lower proportion of Bacteroidetes and a markedly increased proportion of Proteobacteria. Moreover, the HGD and HFrD groups had increased gut permeability due to alterations to the tight junction proteins caused by gut inflammation. Hepatic inflammation and lipid accumulation were also markedly increased in the HGD and HFrD groups. High levels of glucose or fructose in the diet regulate the gut microbiota and increase intestinal permeability, which precedes the development of metabolic endotoxemia, inflammation, and lipid accumulation, ultimately leading to hepatic steatosis and normal-weight obesity.

## 1. Introduction

Obesity is now recognized as a global public health issue, as its prevalence is consistently increasing in most countries [1]. One of the metabolic disorders, obesity, plays an important role in pathogenesis of chronic diseases and is characterized by low-grade inflammation [2]. Sugar consumption has dramatically increased in the past few decades [3] due to the wide availability of convenient, high-sugar foods, as well as an abundance of environmental food cues that prime eating behavior [4]. Overconsumption of sugar is closely linked to obesity and metabolic disease [5]. Thus, the treatment of obesity and its complications has become a major public health focus, and novel treatment strategies would be highly beneficial.

The microbial community within the gut has been linked with several metabolic diseases, including diabetes, non-alcoholic fatty liver disease, cardiovascular disease, and obesity [6,7]. In particular, many studies suggest that the intestinal microbiota play a role in determining body weight [8,9]. Genetically obese ob/ob mice have reduced Bacteroidetes and increased Firmicutes abundance compared with C57BL/6J mice [10]. Moreover, germ-free mice are resistant to the obesogenic effects of a high-fat diet (HFD) [11], whereas transplantation of the gut microbiota from obese mice into germ-free mice recapitulated the donor phenotype [12]. These observations suggest that body weight is influenced by the gut microbiota.

Diet is one of the various factors that influences the microbiota [13]. High levels of fat in the diet change the gut microbial community, particularly by increasing the ratio of Firmicutes to Bacteroidetes [14]. Diet-induced changes in the gut microbiota increase the plasma concentration of the inflammatory bacterial lipopolysaccharide (LPS), which ultimately leads to insulin resistance and glucose intolerance [15]. Moreover, gut microbiota-derived LPS induces inflammation and related metabolic diseases [16]. HFD-induced changes in the gut microbial community enhance intestinal permeability and promote the leakage of LPS into circulation by decreasing the expression of intestinal tight junction proteins [17]. Therefore, bacterial-derived LPS reaches the liver by the portal circulation, inducing inflammation and abnormal lipid accumulation in several tissues, due to increased levels of inflammatory cytokines, such as tumor necrosis factor alpha (TNF-α), monocyte chemoattractant protein 1 (MCP1), interleukin 1 beta (IL-1β), and interleukin 6, and lipid synthesis enzymes, such as acetyl-CoA carboxylase 1, fatty acid synthase (FAS), and sterol regulatory element-binding protein 1 (SREBP1) [18,19,20].

High-sugar consumption induced changes in gut microbiota, obesity and metabolic disorder are well-known [5], but few studies have reported that high-dose fructose or glucose intake correlates with detrimental health outcomes [21]. Crescenzo et al. reported that obesity and insulin resistance are elicited by a high-fructose diet (HFrD) in adult rats [22]. Moreover, a diet high in fructose induces inflammation and metabolic dysregulation in the gut and liver due to alterations in gut microbial communities [23]. However, there is only limited research on high-glucose diet (HGD) or HFrD-induced changes to gut microbiota and the subsequent effects on metabolic diseases. Therefore, the aim of this study was to examine the effects of diets high in monosaccharides on gut microbial diversity, gut permeability, metabolic endotoxemia, and lipid metabolism in C57BL/6J mice.

## 2. Materials and Methods

### 2.1. Materials

A 4-kDa fluorescein isothiocyanate (FITC)-dextran and a phosphatase inhibitor cocktail were purchased from Sigma-Aldrich (St. Louis, MO, USA). The antibodies against TNF-α, IL-1β, MCP1, toll like receptor 4 (TLR4), tight junction protein-1 (ZO1), occludin, FAS, cluster of differentiation 36 (CD36), and SREBP1 were obtained from Abcam (Cambridge, MA, USA). Secondary antibodies were purchased from Thermo Fisher Scientific (Waltham, MA, USA).

### 2.2. Animals and Diets

Six-weeks-old male C57BL/6J mice were purchased from Central Lab Animal Inc. (Seoul, Korea) and were housed at 23 °C in a humidity-controlled (65%) animal room with a 12-h light/12-h dark cycle and provided with food and water ad libitum. The animal experiments were approved by the Animal Welfare Committee of the Korea Food Research Institute (KFRI-M-17045). Eight-week-old mice were assigned to 4 groups (*n* = 9), which were fed a normal diet (ND), HGD, HFrD, or HFD for 12 weeks, and 3 mice were placed in each cage.

The ND (Teklad Global 2018S, Harlan, Madison, WI, USA) contained 18.0% of calories in fat, 24.0% of calories in protein, and 58.0% of calories in carbohydrate. The HFD contained 61.2% of calories in fat (8.8% from soybean oil and 91.2% from lard), 18.8% of calories in protein (98.5% from casein and 1.5% from l-cysteine), and 20.0% of calories in carbohydrate (36% from sucrose and 64% from maltodextrin), and the HGD and HFrD contained 16.9% of calories in fat (8.8% from soybean oil and 91.2% from lard), 18.1% of calories in protein (97.5% from casein and 2.5% from l-cysteine), and 65.0% of calories in carbohydrate (85% from glucose or fructose and 15% from sucrose). Food consumption and weight gain were recorded twice per week until the end of the study.

### 2.3. Oral Glucose Tolerance Tests and Insulin Tolerance Tests

Oral glucose tolerance tests (OGTTs) was performed after 12 h fasting at 4, 8, and 12 weeks. Glucose was orally administered (1 g/kg body weight) and blood glucose levels measured with a glucometer (Accu-Chek^®^, Roche Diagnostics, Indianapolis, IN, USA) at 0, 30, 60, 90, and 120 min after glucose administration. Insulin tolerance tests (ITTs) were performed using human insulin at 4, 8, and 12 weeks (Sigma-Aldrich, St. Louis, MO, USA). The mice were injected insulin intra-peritoneally (1 U/kg) after 4–5 h of fasting. The blood glucose levels were measured at 0, 30, 60, 90, and 120 min after insulin administration.

### 2.4. Intestinal Permeability

FITC-dextran was used to measure the intestinal permeability at 12 weeks, as previously described [24]. Briefly, mice were fasted for 6 h, then administered FITC-dextran by oral gavage (500 mg/kg body weight, 125 mg/mL). One-hundred microliters of blood were collected from the tail vein after 1 h and 4 h. The blood was centrifuged at 12,000× *g* for 5 min at 4 °C. The plasma dextran concentration was measured with a microplate reader (Molecular Devices, Sunnyvale, CA, USA) at an excitation wavelength of 485 nm and emission wavelength of 535 nm. Standard curve was created by diluting FITC-dextran in non-treated plasma diluted with phosphate-buffered saline (1:1, *v*/*v*).

### 2.5. Gut Microbiota Analysis

Fresh fecal samples of mice were collected at week 12 and immediately stored at −80 °C until processing. For microbial community analysis, fecal DNA extraction and V3–V4 hypervariable region of the 16S rRNA gene amplification were carried out using a MiSeq (Illumina, San Diego, CA, USA) at Macrogen (Seoul, Korea) according to the manufacturer’s instructions.

Paired-end reads were assembled using FLASH [25]. Clustering of 16S rRNA operational taxonomic units (OTUs) were defined at ≥97% sequence homology using CD-HIT-OUT and identified using rDnaTools based on reference dataset from the Ribosomal Database Project [26]. And then taxonomic composition was assigned using QIIME-UCLUST [27]. To measure diversities, QIIME software was used based on weighted and unweighted Unifrac distance matrices [28]. Principal coordinate analysis plots and Unweighted Pair Group Method with Arithmetic mean cluster were visualized using XLSTAT software (Addinsoft^®^, New York, NY, USA) and cladograms were produced using GraPhlAn [29].

### 2.6. Blood Serum Analysis

After 12 weeks, the mice were fasted for 12 h and sacrificed by anesthesia. Blood was collected into endotoxin free microfuge tubes by cardiac puncture and allowed to clot. The blood samples were centrifuged at 3000× *g* for 10 min, then collected the serum, which we froze at −80 °C until biochemical analysis. Serum samples were assayed for levels of total cholesterol, low-density lipoprotein (LDL) cholesterol, and endotoxin. Total cholesterol and LDL cholesterol levels were qualified using a Cholesterol Assay Kit (Abcam, Cambridge, MA, USA), and serum endotoxin levels were analyzed with the Pierce™ LAL Chromogenic Endotoxin Quantitation Kit (Thermo Fisher Scientific, Waltham, MA, USA), according to the manufacturers’ instructions.

### 2.7. Western Blotting

Total proteins were extracted from the liver and colon with PRO-PREP™ (iNtRON Biotechnology, Seongnam, Korea) containing phosphatase inhibitor. Equal amounts of protein (30 μg) were loaded for 10%-sodium dodecyl sulfate polyacrylamide gel electrophoresis, which we then transferred to a membrane. The membranes were blocked with 5% skim milk for 1 h at room temperature (25 ± 2 °C) and incubated with the primary antibodies overnight at 4 °C. In all conditions, primary antibodies were used as 1:1000. Then, the membranes were incubated with peroxidase-labeled secondary antibodies for 1 h at room temperature. Immunoreactive proteins were detected with enhanced chemiluminescence reagents using a ChemiDoc™ XRS+ imaging system (Bio-Rad, Hercules, CA, USA).

### 2.8. Histological Analysis

Histological analyses were performed after hematoxylin and eosin (H&E) staining. Liver and epididymal white adipose tissue (WAT) were fixed in 10% formalin after mice were sacrificed. The fixed tissues were embedded in paraffin and sliced into 5-µm sections. Then, the tissue sections were stained with H&E. We acquired digital images using an optical microscope (Nikon Eclipse Ti-E, Nikon, Kobe, Japan). The hepatic steatosis score was calculated according to the method by Kato et al. [30] (0, none; 1, <33%; 2, 33–66%; 3, >66%) and adipocyte area was determined using Image J software (NIH, Bethesda, MD, USA).

### 2.9. Statistical Analysis

The data are presented as the mean ± standard error of the mean. The statistical significance of the differences among groups were determined by one-way analysis of variance with Tukey’s analysis using GraphPad Prism software (San Diego, CA, USA). A *p*-value < 0.05 was considered statistically significant.

## 3. Results

### 3.1. Effects of Diet on Body Weight and Metabolic Parameters

**Figure 1:**
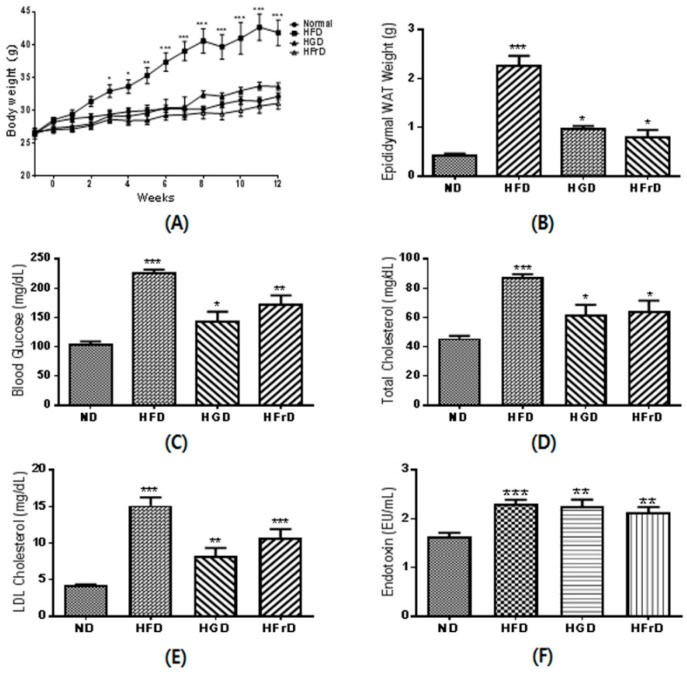
Metabolic disorder parameters in high-glucose diet (HGD) or high-fructose diet (HFrD) fed mice. (**A**) body weight changes during the 12 weeks of feeding; (**B**) white adipose tissue (WAT) weight; (**C**) fasting blood glucose; (**D**) serum total cholesterol; (**E**) serum low-density lipoprotein (LDL)-cholesterol; (**F**) serum endotoxin. Data are presented as the mean ± SEM for 9 mice per group (* *p* < 0.05, ** *p* < 0.01, and *** *p* < 0.001 vs. ND).

At the end point of the experiment, the HFD-fed mice had significantly higher body weights than the ND-fed mice (Figure 1A). As expected based on their increased body weight, the mice in the HFD-fed group also had a markedly higher epididymal WAT mass (Figure 1B). The body weights of the HGD- and HFrD-fed mice were not increased over those of the ND-fed mice, although their WAT masses were significantly increased.

The fasting blood glucose concentration was significantly increased in the HFD, HGD, and HFrD groups (Figure 1C), as well as the total and LDL cholesterol (Figure 1D,E). Moreover, these mice had significantly higher serum endotoxin levels than the ND-fed mice (Figure 1F). These results suggest that not only high levels of fat, but also high levels of glucose or fructose, in the diet induce lipid accumulation and endotoxemia.

### 3.2. Effects of Diet on Gut Microbial Diversity and Composition

**Figure 2:**
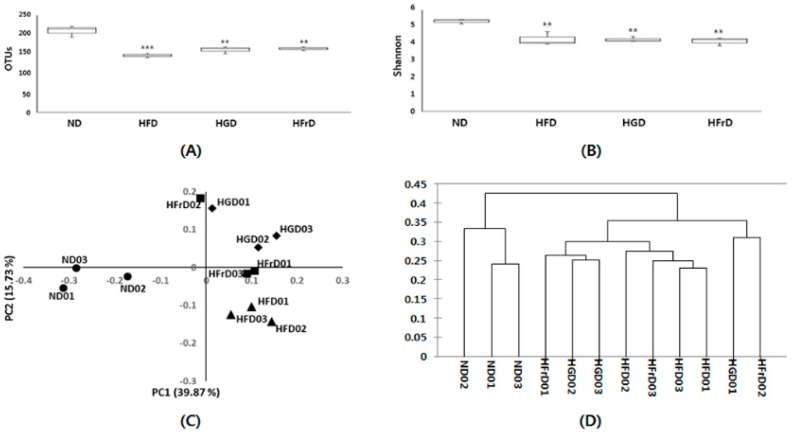

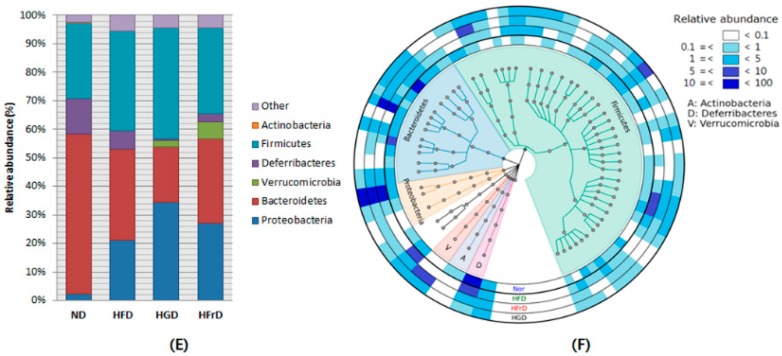
Analysis of the gut microbial community by 16S rRNA pyrosequencing from feces of HFD, HGD and HFrD groups. (**A**) operational taxonomic units levels; (**B**) Shannon’s diversity indices; (**C**) principal coordinate analysis of unweighted UniFrac analysis; (**D**) sample clustering results based on the unweighted UniFrac analysis; (**E**) relative abundances plot of bacterial phyla; (**F**) relative abundance cladogram of bacterial taxa. Data are presented as the mean ± SEM for 3 cages per group (** *p* < 0.01, and *** *p* < 0.001 vs. ND).

Many studies have reported that a high fat concentration in the diet alters the gut microbiota and increases endotoxemia [31,32]. We confirmed this finding by 16S RNA analysis. We observed fewer operational taxonomic units and lower Shannon indices in the HFD, HGD, and HFrD groups than in the ND group (Figure 2A,B). We assessed the phylogenetic differences in the gut microbiota among the groups by principal coordinates analysis. The ND group had a distinct microbial composition that clustered separately from those of the HFD, HGD, and HFrD groups (Figure 2C). We performed hierarchical clustering analysis according to the data matrix of the unweighted pair group method with arithmetic mean; we found that the microbial communities in the feces of the HGD- and HFrD-fed mice were more closely related to those of the HFD-fed mice than those of the ND-fed mice (Figure 2D).

Taxon-based analysis showed marked changes in the gut microbial compositions of the HFD, HGD, and HFrD groups. At the phylum level, these groups had a significantly lower relative abundance of Bacteroidetes and significantly increased abundance of Proteobacteria compared to the ND group (Figure 2E). In particular, the proportions of *Muribaculum intestinale* (phylum, Bacteroidetes) were significantly lower in the HFD-, HGD-, and HFrD-fed mice and the proportions of *Desulfovibrio vulgaris* were increased (phylum, Proteobacteria). Interestingly, we observed a higher proportion of *Akkermansia muciniphila* in the HGD and HFrD groups than in the HFD group (Figure 2F).

### 3.3. Effects of Diet on Glucose Intolerance and Insulin Resistance

**Figure 3:**
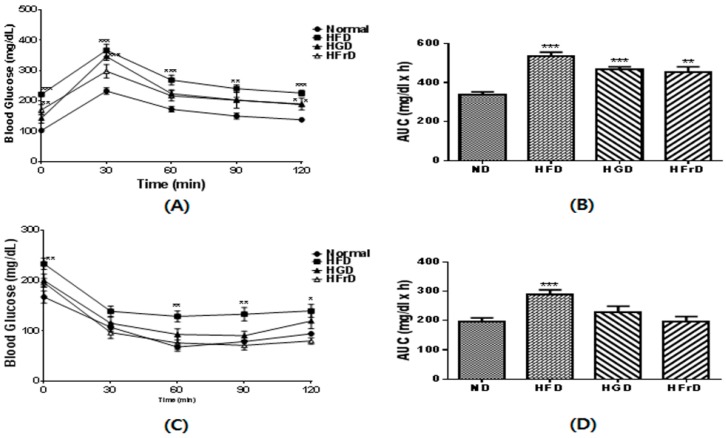
Glucose metabolism in HFD, HGD or HFrD fed mice. (**A**) blood glucose levels during an oral glucose tolerance tests; (**B**) area under the curve (AUC) of blood glucose levels; (**C**) blood glucose levels during an insulin tolerance tests; (**D**) AUC of blood glucose levels. Data are presented as the mean ± SEM for 9 mice per group (* *p* < 0.05, ** *p* < 0.01, and *** *p* < 0.001 vs. ND).

To investigate if the diet-induced microbiota changes were associated with changes in glucose intolerance and insulin resistance in mice, we performed OGTTs and ITTs. As shown in Figure 3A, after 12 weeks HFD-feeding significantly increased blood glucose levels over those in the control mice. Moreover, HGD- and HFrD-fed mice also had significantly increased glucose intolerance (Figure 3A,B). As shown in Figure 3C, we found a higher fasting glucose concentration in the HFD group than in the ND group, after 12 weeks. However, the plasma glucose levels of the HGD and HFrD groups showed similar pattern compared to the ND group after insulin injection (Figure 3C,D).

### 3.4. Effects of Diet on Gut Permeability and Inflammation

**Figure 4:**
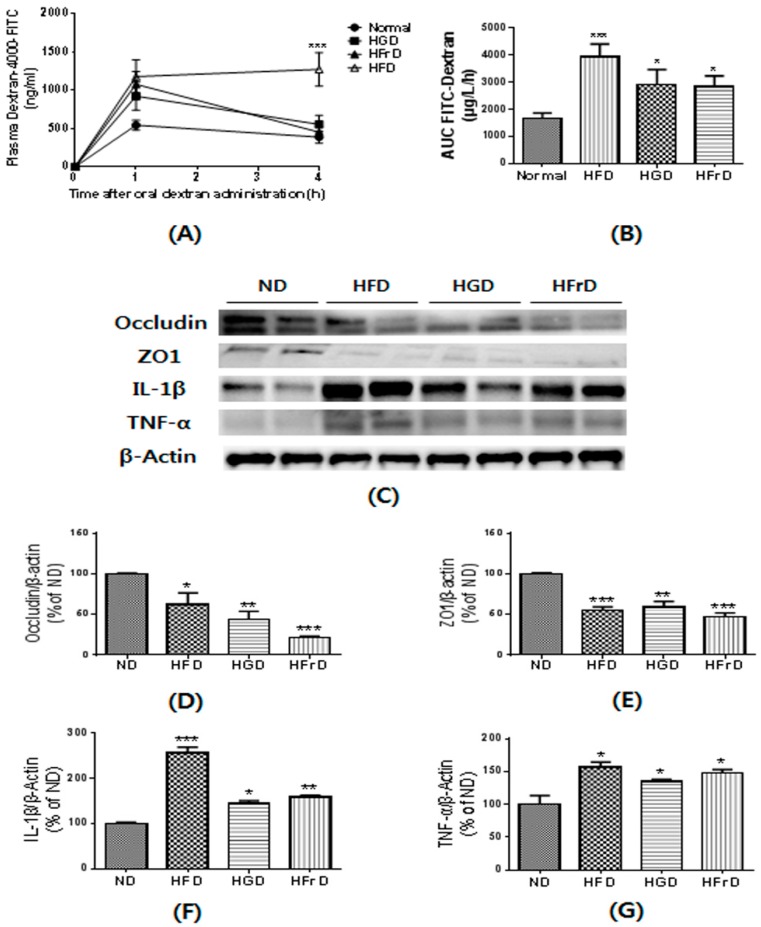
HFD, HGD or HFrD-induced changes of gut permeability and related proteins expression. (**A**) plasma fluorescein isothiocyanate (FITC)-dextran concentration; (**B**) AUC of Plasma FITC-dextran levels; (**C**) representative images of Western blots for tight junction proteins (Occludin and ZO1) and inflammatory cytokines (IL-1β and TNF-α); (**D**–**G**) relative band intensities of Occludin (**D**), ZO1 (**E**), IL-1β (**F**) and TNF-α (**G**) normalized to those of β-actin. Data are presented as mean ± SEM for 9 mice per group (**A**,**B**) and mean percentage of ND ± SEM of three independent experiments (C-G) (* *p* < 0.05, ** *p* < 0.01, and *** *p* < 0.001 vs. ND).

We assessed gut permeability using the paracellular tracer FITC-dextran just prior to the end of the experiment. Following oral administration, HFD-fed mice exhibited a 2.5-fold greater area under the curve for plasma FITC-dextran than the ND-fed mice (Figure 4A,B). The HGD- and HFrD-fed mice also showed significantly higher plasma FITC-dextran levels. Gut permeability is controlled by tight junction proteins, such as ZO-1 and occludin [24]. The HFD, HGD, and HFrD groups had less abundant ZO-1 and occludin expression in the colon than ND mice (Figure 4D,E).

Increased gut permeability induced by diet-induced obesity has been reported to cause metabolic endotoxemia and inflammation [33]. To assess the effects of diet on intestinal inflammation, we investigated the expression of inflammatory cytokines. The HFD, HGD, and HFrD groups had significantly higher expression of inflammatory cytokines, such as TNF-α and IL-1β, in the colon than ND mice (Figure 4F,G). Taken together, these findings indicate that diet-induced changes in the gut microbiota affect the expression of tight junction proteins and inflammatory cytokines, which leads to increased gut permeability and inflammation.

### 3.5. Effects of Diet on Liver Inflammation and Lipid Metabolism

**Figure 5:**
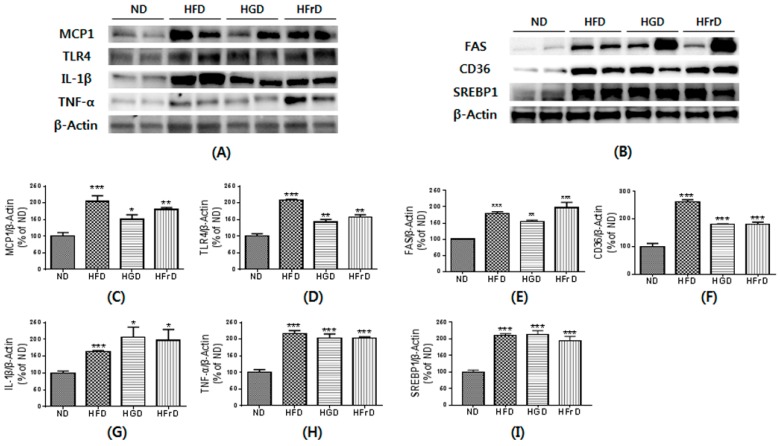
HFD, HGD or HFrD-induced hepatic inflammation and change of lipid metabolism. (**A**,**B**) representative images of Western blots for inflammatory cytokines (MCP1, TLR4, IL-1β and TNF-α) and lipid metabolism (FAS, CD36 and SREBP1); (**C**–**I**) relative band intensities of MCP1 (**C**), TLR4 (**D**), IL-1β (**E**), TNF-α (**F**), FAS (**G**), CD36 (**H**) and SREBP1 (**I**) normalized to those of β-actin. Bar values are presented as mean percentage of ND ± SEM of three independent experiments (* *p* < 0.05, ** *p* < 0.01, and *** *p* < 0.001 vs. ND).

Increased endotoxemia can induce liver inflammation [34]. To link the changes in the gut microbiota to diet-induced markers of metabolic disease, we assessed the expression of inflammatory cytokines. As shown in Figure 5A, the protein expression of MCP1, TLR4, IL-1β, and TNF-α in the liver were quantified to evaluate hepatic inflammation. As expected, inflammatory cytokines were significantly increased in the HFD-fed mice over their levels in the ND-fed mice. The HGD- and HFrD-fed mice also had markedly increased inflammatory cytokine expression. These changes suggest strong relationships among the gut microbiota, gut permeability, and tissue inflammation in a diet-induced mouse inflammation model.

Hepatic inflammation is normally accompanied by hepatic lipid accumulation. We analyzed the expression of regulatory proteins involved in lipid metabolism, such as FAS, CD36, and SREBP1, in the liver (Figure 5B). As expected, HFD-fed mice exhibited increased expression of these proteins. Interestingly, HGD- and HFrD-fed mice also significantly increased the FAS, CD36 and SREBP1 to a level similar to the ND group. These results suggest that the HGD and HFrD up-regulate lipid metabolism-related protein expression, thereby contributing to hepatic steatosis.

### 3.6. Histological Changes

**Figure 6:**
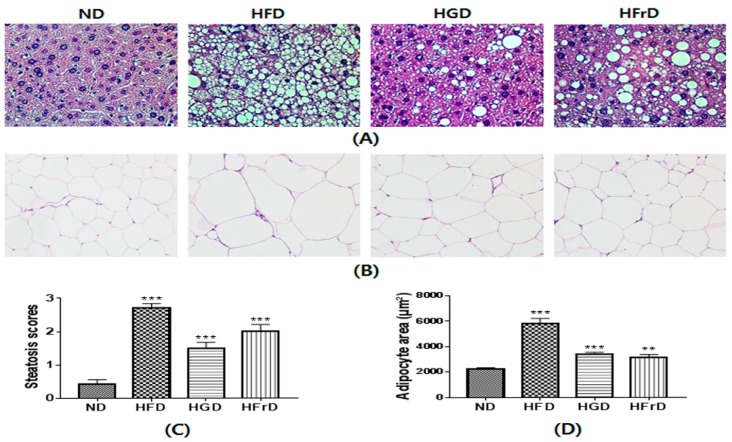
HFD, HGD or HFrD-induced hepatic steatosis and adipocyte hypertrophy. (**A**,**B**) representative histological results of liver and WAT by hematoxylin and eosin staining; (**C**) steatosis score of liver; (**D**) quantification of adipocyte area. Values are expressed as means ± SEM for 3 mice per group (** *p* < 0.01, and *** *p* < 0.001 vs. ND).

We confirmed the development of hepatic steatoses in H&E-stained liver sections (Figure 6). As expected, the HFD induced severe hepatic lipid accumulation. The HGD and HFrD also increased lipid deposition in the liver compared to the level caused by the ND (Figure 6A).

Histological analysis of WAT showed that the increase in body weight was associated with an increase in the size of the adipocytes in the HFD mice (Figure 6B). There were no significant changes in body weight between the ND mice and the HGD or HFrD mice; however, steatosis scores and the size of adipocytes were markedly increased in the HGD and HFrD mice (Figure 6C,D). These results suggest that the HGD and HFrD can cause hepatic steatosis and obesity in the absence of weight gain.

## 4. Discussion

The phylogenetic and metagenomic analysis of gut microbiota have been extensively studied in the context of metabolic disorders. HFD-induced inflammation and metabolic disorders are clearly linked to changes in gut microbiota [35,36]. A long-term HFD results in obesity, lipid accumulation, and dyslipidemia [37]. Moreover, glucose intolerance and insulin resistance can result from HFD-induced obesity [38]. Our findings supported the published findings on the effects of a HFD. We also found that high glucose and fructose levels in the diet had similar effects in the absence of elevated body weight. Although glucose tolerance differed between the ND-fed mice and those on the modified diets, only the HFD-fed mice were clearly insulin-resistant, suggesting that increased fasting blood glucose levels, dyslipidemia, and glucose intolerance may be caused by increased endotoxin levels.

Several lines of evidence suggest that gut microbiota play an important role in obesity and associated disorders, such as dyslipidemia, inflammation, and glucose intolerance [39,40]. A diet that is high in fat can reshape the gut microbiota, particularly by increasing the Firmicutes-to-Bacteroidetes ratio [41]. Moreover, the proportion of Proteobacteria, which are known to be among the best sources of LPS, is increased in HFD-fed mice [42]. Jang et al. reported that low doses of fructose are cleared by the small intestine, but high doses of fructose are digested by microbiota and liver and one of the possible mechanism of fatty liver is the conversion of fructose into a hepatotoxic metabolite [43]. Based on these observation, we hypothesized that high-dose monosaccharides were not cleared by the small intestine, thereby changing the gut microbiota and inducing metabolic disorder. In the present study, we observed increased Firmicutes-to-Bacteroidetes ratios and widespread changes in gut microbial communities, including increased proportions of Proteobacteria and decreased proportions of Actinobacteria in the HFD, HGD, and HFrD groups compared to in the ND group. These results indicate that the HGD and HFrD, as well as the HFD, modulate gut microbiota and cause gut microbiota-induced inflammation and fatty liver.

An altered gut microbiota composition can increase the level of the Gram-negative bacterial product LPS [44]. Rainone et al. demonstrated that obesity in children and adolescents is characterized by up-regulation of LPS and subsequent inflammation [45]. Recently, researchers have proposed that high levels of endotoxemia are related to gut permeability and decreased tight junction protein expression such as ZO1 and occludin [16,46]. In this study, we confirmed that HFD-fed mice exhibit increased gut permeability and an altered gut barrier, characterized by disruption of the tight junction proteins. Moreover, we found that the HGD and HFrD also increased gut permeability and disrupted the gut barrier. The damaged gut barriers observed in HFD-, HGD-, and HFrD-fed mice correlate with higher plasma endotoxin levels. Among the mechanisms involved in this phenomenon, over-expressed TNF-α is known to increase local or systemic inflammation, which can trigger alterations in tight junction proteins [47]. In this study, we found increased expression of the tight junction-disrupting cytokines TNF-α and IL-1β in the colons of the HFD-, HGD-, and HFrD-fed mice. Together, these data suggest that diet-induced changes in the gut microbiota can cause endotoxemia and colon inflammation, thereby damaging the intestinal barrier and contributing to metabolic disorders.

Intestinal inflammation-induced leakage of gut microbiota-derived endotoxin is a potent inducer of hepatic steatosis, characterized by abnormal fat deposition in the liver [46]. Hepatic lipid accumulation upregulates pro-inflammatory cytokines and apoptotic signals in liver through directly activating the TLR-4 pathways [48]. Hence, we measured the protein levels of factors associated with inflammation and lipid metabolism in the liver. The HFD, HGD, and HFrD caused marked increases in TNF-α, IL-1β, TLR-4, and MCP1 expression. These results suggest that the HGD and HFrD can induce hepatic inflammation via gut microbiota-derived endotoxin. Furthermore, we found markedly increased CD36 expression in the HFD, HGD, and HFrD groups. CD36 plays an important role in promoting hepatic fat uptake and triglyceride storage in HFD-fed mice [49]. Moreover, mice in the HFD, HGD, and HFrD groups exhibited increased levels of FAS and SREBP1 compared with the levels in the ND group. FAS is a key enzyme in lipogenesis, which may play an important role in the pathogenesis of hepatic steatosis through fatty acid synthesis [50]. SREBP1 promotes lipogenesis via regulation of the fatty acid biosynthesis enzymes [51]. Thus, the higher inflammatory cytokine expression observed in the HFD-, HGD-, and HFrD-fed mice could induce up-regulation of CD36, FAS, and SREBP1, thereby contributing to the development of a fatty liver.

We confirmed the abnormal accumulation of fat in the liver induced by enhanced lipid metabolism and inflammatory cytokine expression by H&E staining. As expected, fat deposition was markedly increased in the HFD-, HGD-, and HFrD-fed mice. The effects of diet-induced changes in the intestinal microbial community were observed not only in the colon and liver, but also in the adipose tissue. Our H&E staining revealed markedly larger adipocytes in the mice on the modified diets. This phenomenon is also reportedly caused by inflammation and altered lipid metabolism downstream of excessive gut microbiota-induced endotoxin release [52]. These findings suggested that HGD- or HFrD-induced increase of gut microbiota-derived endotoxin and the pathogenesis of fatty liver were closely linked, evidencing a key role for the gut microbiota as regulator of the gut-liver axis.

In this study, we revealed that a diet high in glucose or fructose can induce changes in gut microbiota, gut permeability, inflammation, hepatic steatosis, and lipid accumulation. It is well-known that high-sugar diets promote obesity [53], however the present study showed high-monosaccharide diet groups did not change the body weight compared with ND group. The HGD and HFrD promoted an increased abundance of Akkermansia compared to the HFD group (Figure 2F), which reduces body weight and improves body composition without changes in food intake [54]. Thus, we attribute the normal body weight of the HGD- and HFrD-fed mice to the increased abundance of Akkermansia. Currently, normal-weight obesity in Asians may play an important role in the development of metabolic complications [55,56]. We propose that the increase in the prevalence of normal-weight obesity is caused by high levels of monosaccharides in the body due to a carbohydrate-rich diet.

In summary, we have demonstrated that the modulation of gut microbiota is associated with increased intestinal permeability, which coincides with the development of metabolic endotoxemia, inflammation, and lipid accumulation in HGD- and HFrD-fed mice, ultimately leading to hepatic steatosis and normal-weight obesity. Further studies should be undertaken to determine the mechanisms that regulate obesity induced by a diet high in glucose or fructose.
